# Predictors for De Novo Overactive Bladder after Readjustable Mid-Urethral Sling Procedure in Women with Stress Urinary Incontinence due to Intrinsic Sphincter Deficiency

**DOI:** 10.1155/2018/6934747

**Published:** 2018-11-26

**Authors:** Joo Hyun No, Kyung Hwa Choi, Dae Keun Kim, Tae Heon Kim, Seung Ryeol Lee

**Affiliations:** ^1^Department of Urology, CHA Bundang Medical Center, CHA University, Seongnam, Republic of Korea; ^2^Department of Urology, CHA Seoul Station Fertility Center, CHA University, Seoul, Republic of Korea

## Abstract

**Purpose:**

This study identified noninvasive factors that predict overactive bladder (OAB) after readjustable mid-urethral sling surgery (Remeex system) in women with stress urinary incontinence (SUI) due to intrinsic sphincter deficiency (ISD).

**Materials and Methods:**

We retrospectively reviewed the medical records of 130 women with SUI due to ISD [Valsalva leak-point pressure (VLPP) <60 cm H_2_O] who underwent the Remeex procedure between February 2011 and March 2017. Patients were classified according to OAB symptoms before and 6 months after the Remeex procedure: Group 1, without preoperative and postoperative OAB (*n*=46); Group 2, without preoperative OAB and with postoperative OAB (de novo OAB,* n*=15); Group 3, with preoperative OAB and without postoperative OAB (*n*=25); Group 4, with preoperative and postoperative OAB (*n*=44). Noninvasive clinical and urodynamic factors were evaluated as predictors of de novo OAB.

**Results:**

The four groups significantly differed with respect to age (*p*=0.036), peak urinary flow rate (PUFR) one month after surgery (post-PUFR,* p*=0.001), and postvoid residual (PVR) one month after surgery (post-PVR,* p*=0.005). No significant differences were detected for body mass index, diabetes, multiparity, menopause, previous hysterectomy, previous incontinence surgery, previous pelvic organ prolapse surgery, pyuria, preoperative PUFR, preoperative PVR, maximal cystometric capacity, VLPP, maximum urethral closure pressure, detrusor pressure at PUFR, and detrusor overactivity (*p*>0.05). Post-PUFR decreased significantly compared with preoperative PUFR in Groups 1, 2, and 4 (*p*=0.002,* p*=0.001, and* p*=0.001, respectively). Pairwise comparisons of post-PUFR and post-PVR revealed statistically significant differences between Group 2 and other groups (*p*<0.0125). Multivariate logistic regression analyses showed that post-PUFR was the only significant predictor of de novo OAB (odds ratio = 0.823, 95% confidence interval 0.727-0.931,* p*=0.002).

**Conclusions:**

Reduced PUFR after the Remeex procedure is a promising predictor of risk for de novo OAB. This metric is noninvasive and easy to measure.

## 1. Introduction

Patients with stress urinary incontinence (SUI) display a spectrum of urethral characteristics ranging from a highly mobile urethra with good intrinsic function to an immobile urethra with poor intrinsic function [1]. The definition of urethra with poor intrinsic function is imprecise [2, 3], but this condition is considered to have intrinsic sphincter deficiency (ISD), which is recognized as a risk factor for failure of the mid-urethral sling procedure [4–6]. The 12-month success rates of the mid-urethral sling procedure in 72 patients with SUI were 91% in the tension-free vaginal tape (TVT) group and 89% in the transobturator tension-free vaginal tape (TVT-O) group [7]. The 5-year objective cure rate of 254 patients with SUI was 84.7% in the TVT group and 86.2% in the TVT-O group [8]. By contrast, ISD patients have a 6-month cure rate of only 79% in the TVT group and 55% in the TVT-O group [9].

The readjustable mid-urethral sling (Remeex system; Neomedic International, Terrassa, Spain) has the advantages of regulating of sling tension postoperatively and avoiding urinary obstruction or persistent SUI due to inappropriate sling tension [10]. The Remeex procedure has good efficacy even in ISD patients. Among 102 women with previous failed surgery or ISD, 91 (89%) patients were cured and 6 (6%) were improved at 27 months of mean follow-up [11]. Sling tension readjustment was needed in 14 patients (14%). Among 50 SUI patients with ISD, 45 (90%) patients were cured and 3 (6%) were improved at 7 years of mean follow-up [12]. Sling tension readjustment was needed in three patients (6%).

Urinary obstruction or persistent SUI is no longer serious conditions since the readjustable mid-urethral sling was introduced; however, de novo overactive bladder (OAB) or worsening preexistent OAB symptoms continue to be challenging. These symptoms may reduce patient satisfaction after the mid-urethral sling procedure and adversely affect health-related quality of life more than other forms of urinary incontinence [13]. Postoperative urinary tract infections, bladder outlet obstruction, urinary tract perforation, and idiopathic urgency have been suggested as possible causes of de novo OAB after sling surgery. The aim of this study is to identify noninvasive clinical parameters that can be used to predict OAB in women with SUI due to ISD following the readjustable mid-urethral sling surgery.

## 2. Materials and Methods

### 2.1. Patients

**Figure 1:**
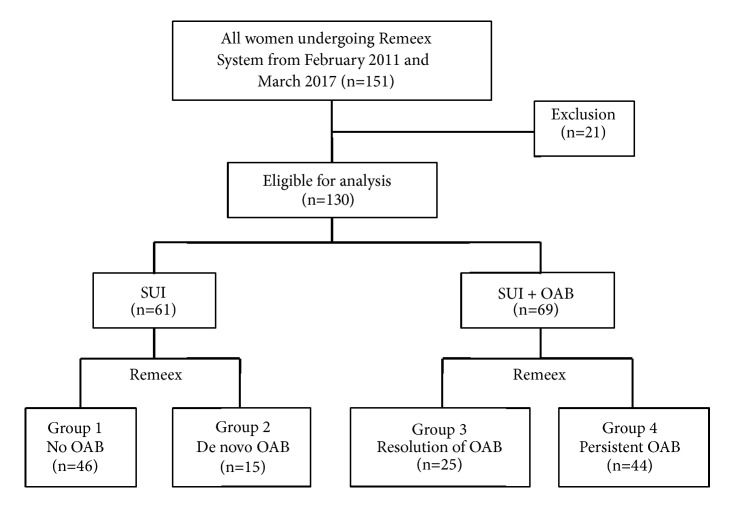
**Study cohort and distribution of women according to the presence of overactive bladder (OAB) symptoms before and 6 months after the Remeex procedure**. Group 1, without preoperative and postoperative OAB; Group 2, without preoperative OAB and with postoperative OAB; Group 3, with preoperative OAB and without postoperative OAB; Group 4, with preoperative and postoperative OAB.

We obtained approval for this study from the Institutional Review Board at CHA Bundang Medical Center (approval number: 201810038). The medical records of 151 women with SUI due to ISD who underwent the Remeex procedure between February 2011 and March 2017 were reviewed. The following inclusion criteria were used: (1) aged ≥18 years, (2) Valsalva leak-point pressure (VLPP) <60 cm H_2_O measured by urodynamic studies, and (3) more than 1 year of follow-up after readjustable mid-urethral sling surgery. A total of 130 patients met the inclusion criteria (Figure 1). Exclusion criteria included the presence of lower urinary tract pathology such as urinary tract calculus, bladder tumors, interstitial cystitis, clinically significant bladder outlet obstruction, and symptomatic or recurrent urinary tract infections. Subjects who had a neurogenic cause underlying OAB also were excluded.

Women with SUI due to ISD were classified into one of the following four groups according to the presence of OAB symptoms before and 6 months after the Remeex procedure: Group 1, without preoperative and postoperative OAB (*n*=46); Group 2, without preoperative OAB and with postoperative OAB (de novo OAB,* n*=15); Group 3, with preoperative OAB and without postoperative OAB (*n*=25); Group 4, with preoperative and postoperative OAB (persistent OAB,* n*=44) (Figure 1).

### 2.2. Preoperative Examination

The preoperative examination included a detailed medical and surgical history, physical examination, a 3-day bladder diary using a 5-point urgency rating scale, urine analysis and culture, stress test, and urodynamic study, which included the maximal cystometric capacity, VLPP, maximum urethral closure pressure (MUCP), detrusor pressure at peak urinary flow, uroflowmetry, and postvoiding residual measurement. The results of uroflowmetry were accepted when voided urine volume was more than 150 ml, PUFR was measurable, and an adequate voiding curve was generated. Women who did not void greater than 150 ml were asked to repeat the test after drinking water. ISD was defined as VLPP <60 cm H_2_O. Women who displayed urinary frequency (≥8 voids/24 h), urinary urgency (≥6 episodes/3 d), or urge incontinence (≥3 episodes/3 d) were considered as OAB patients.

### 2.3. Surgical Procedures

The readjustable mid-urethral sling surgery was performed using the Remeex system. The Remeex device consisted of a suburethral polypropylene prosthesis that was linked to a pressure adjusting device (varitensor) by two traction threads. The varitensor was implanted permanently in the abdominal rectus muscle fascia, and the postoperative sling tension was adjusted by connecting the manipulator to the varitensor.

The surgical procedure was performed under spinal anesthesia with the patient placed in the dorsal lithotomy position. A 4 cm abdominal transverse incision was made 2 cm above the symphysis pubis, and the dissection was continued until the rectus sheath was exposed. The anterior vaginal wall was incised from the middle urethra to the urethrovesical junction (approximately 2 cm) and then dissected from the underlying periurethral tissues to the endopelvic fascia. A needle was passed through the retropubic space to perforate the abdominal muscle fascia at the lateral margins of the transverse incision from the vaginal to the abdominal plane. A cystoscopy was performed to ensure that the bladder had not been perforated. Then, the traction threads were passed through a needle-eye and drawn upward on each side until it appeared at the abdominal incision. A polypropylene mesh was placed at the mid-urethral level. The traction threads were inserted into the varitensor and knotted together. The manipulator was then rotated clockwise until the varitensor lay on the rectus sheath without tension. The vaginal and abdominal incisions were closed with the manipulator protruding through the abdominal incision.

Patients were examined the day after surgery. They were asked whether they could urinate without any difficulty, and they were asked to perform a cough test or any activity that would generally result in SUI. If there was a urine leak, the manipulator was rotated to tighten the sling until no further leakage occurred without significant residual urine. The manipulator was removed after the patient had reached continence.

### 2.4. Follow-Up

Follow-up visits were scheduled at 1, 3, 6, and 12 months, and then every 12 months thereafter. Each follow-up examination included uroflowmetry, postvoid bladder scanning, and a stress test to measure the degree of incontinence. If urine leak was detected, the Remeex system was adjusted as follows. The patient was placed under local anesthetic, the manipulator was reattached to the varitensor through a previous abdominal incision, and the sling tension was readjusted. A 3-day bladder diary using a 5-point urgency rating scale was performed at 6 months after the surgery.

### 2.5. Statistical Analysis

The following nine clinical parameters were evaluated as potential predictors of de novo OAB: age, body mass index (BMI), diabetes, multiparity, menopause, previous hysterectomy, previous incontinence surgery, previous pelvic organ prolapse surgery, and pyuria. The following nine urodynamic parameters were evaluated as potential predictors of de novo OAB: preoperative peak urinary flow rate (pre-PUFR), preoperative postvoid residual (pre-PVR), maximal cystometric capacity (MCC), VLPP, MUCP, detrusor pressure at peak urinary flow (PdetQmax), detrusor overactivity (DO), peak urinary flow rate one month after surgery (post-PUFR), and postvoid residual one month after surgery (post-PVR). The potential predictive factors were compared among the four groups using the Kruskal-Wallis test and Chi-square test. To identify significant factors that affect de novo OAB, univariate and multivariate logistic regression analyses were performed. Regression analysis results are presented as odds ratio (OR) and 95% confidence interval (CI). Statistical analyses were performed using SPSS 24.0 (IBM Corp., Armonk, NY). Data are presented as the mean ± standard deviation. A* p* value less than 0.05 was considered as statistically significant.

## 3. Results

**Figure 2:**
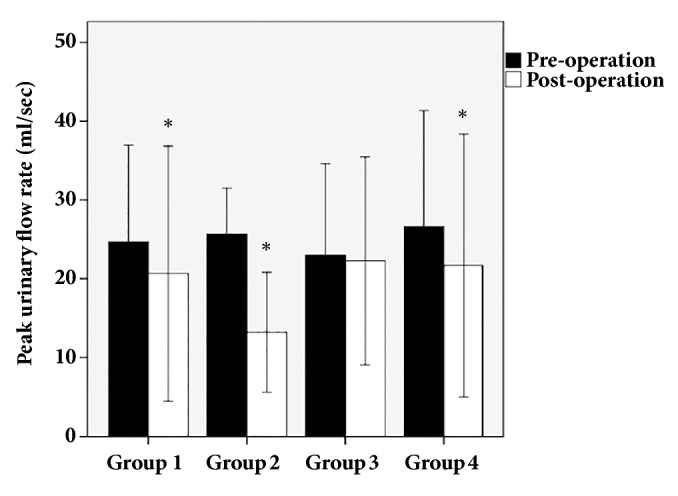
**Comparison of changes in peak urinary flow rate (PUFR) between the four groups before and 1 month after the Remeex procedure**. The post-PUFR decreased significantly compared with pre-PUFR in Groups 1, 2, and 4. *∗p* <0.05.

The study enrolled 130 patients. The mean age was 59 ± 11 years (range 33-86 years, Table 1). The four groups differed significantly with respect to age (*p*=0.036), post-PUFR (*p*=0.001), and post-PVR (*p*=0.005, Table 1). By contrast, there were no significant differences among the four groups with respect to BMI, diabetes, multiparity, menopause, previous hysterectomy, previous incontinence surgery, previous pelvic organ prolapse surgery, pyuria, pre-PUFR, pre-PVR, MCC, VLPP, MUCP, PdetQmax, and DO (*p*>0.05). The post-PUFR decreased significantly compared with pre-PUFR in Groups 1, 2, and 4 (*p*=0.002,* p*=0.001, and* p*=0.001, respectively) (Figure 2). There were no significant differences between pre-PUFR and post-PUFR in Group 3 (p=0.269).

Pairwise comparisons of age, post-PUFR, and post-PVR indicated that post-PUFR and post-PVR significantly differed between Group 2 and the other groups (*p*<0.0125, Table 2). Post-PUFR was significantly lower in Group 2 than in other groups. Post-PVR was also significantly higher in Group 2 than in other groups.

Table 3 presents the results of univariate and multivariate logistic regression analyses of clinical and urodynamic factors as predictors of de novo OAB. Among 130 women with SUI due to ISD, 15 patients had de novo OAB. Multivariate logistic regression analyses indicated that post-PUFR was the only significant predictor of de novo OAB after the Remeex procedure (OR = 0.823, 95% CI 0.727-0.931,* p*=0.002). Multivariate analyses indicated that age and post-PVR were not predictive of de novo OAB.

## 4. Discussion

We found that post-PUFR decreased significantly after surgery with the Remeex system, and the post-PUFR of Group 2 (de novo OAB) had the largest decrease compared with the other three groups. Statistical analyses indicated that the post-PUFR decrease was significantly associated with de novo OAB after surgery with the Remeex system. We anticipated this result because obstruction of the bladder outlet was hypothesized as the possible cause of de novo OAB. Other possible causes of de novo OAB after anti-incontinence surgery include postoperative urinary tract infection and foreign bodies such as mesh or suture materials with or without adherent calculus [14]. Among these possible causes, bladder outlet obstruction could alter receptor function, myogenic denervation, and neurotransmitter balance, leading to detrusor overactivity [15].

Although decreased post-PUFR does not correspond exactly with bladder outlet obstruction, maximum urinary flow rate ≤15 ml/sec appeared to be the most discriminating parameter of female bladder outlet obstruction in neurologically intact women [16]. Patients in Group 2 were considered to have bladder outlet obstruction after the Remeex procedure because their mean PUFR decreased from 25.7 ml/sec to 13.2 ml/sec. Mean post-PUFR was <15 ml/sec in Group 2 after the Remeex procedure. The bladder outlet obstruction index (BOOI) was developed to diagnose benign prostatic obstruction in older men [17]. BOOI can be calculated from PdetQmax and PUFR, which are measured by urodynamic studies. However, to obtain BOOI, the urodynamic study should be performed again after mid-urethral sling surgery. Some patients feel discomfort throughout the urodynamic study. In a survey to query patient responses to the urodynamic study, of 314 patients who completed the questionnaire (60% response rate), 29.0% and 12.4% of respondents reported physical and emotional discomfort, respectively, although half of the respondents did not feel discomfort [18]. Patients who had undergone previous anti-incontinence surgery reported significantly higher pain levels during the urodynamic study [19]. Based on these data, we consider routine follow-up urodynamic study to be unreasonable and invasive, and recommend noninvasive methods to assess poor urinary stream. Therefore, the measurement of urinary flow rate provides a promising metric to screen for de novo OAB because it is noninvasive and easy to perform.

Several possible predictors associated with de novo OAB symptoms after mid-urethral sling procedure have been investigated. Lee et al. reported that ISD, previous stress incontinence surgery, concomitant apical prolapse operation, previous prolapse surgery, and preexisting DO were important predictors of de novo OAB symptoms [20]. Marcelissen and Van Kerrebroeck identified the following risk factors of OAB symptoms after mid-urethral sling surgery in women: urgency, use of anticholinergic medications, previous incontinence surgery, older age, and urodynamic signs of OAB such as DO, lower bladder capacity, and elevated detrusor pressure [21]. In the present study, these aforementioned predictors did not significantly differ among the four patient groups, possibly because our subjects were ISD patients in whom reduced PUFR was the only parameter significantly associated with de novo OAB. Detrusor pressure on voiding was significantly lower in ISD patients than in non-ISD patients. Therefore, the effect of bladder outlet obstruction on de novo OAB may be more significant in ISD patients after the Remeex procedure [22].

It is important to determine when OAB symptoms should be evaluated because lower urinary tract symptoms that arise after mid-urethral sling surgery often disappear with increasing time after surgery [23, 24]. Liang et al. reported that most OAB symptoms resolved without intervention by 3 months after surgery in patients treated with transobturator sling procedures [23]. Rechberger et al. reported that the majority of undesired lower urinary tract symptoms spontaneously resolved within the first 6 months after mid-urethral sling surgery. In general, the number of urgency episodes significantly declined by 6 months after surgery compared with baseline. Therefore, we evaluated de novo OAB symptoms during the 6-month follow-up examination after the Remeex procedure.

This study has some limitations. First, this was a retrospective study, so we did not perform postoperative urodynamic tests to confirm the bladder outlet obstruction. However, we think that it will be possible to distinguish female bladder outlet obstruction from neurologically intact women using the parameter of maximum urinary flow rate ≤15 ml/sec instead of the urodynamic study. Second, although post-PUFR decrease after the Remeex procedure was the most prominent predictor in the de novo OAB group (Group 2), it was not easy to obtain good cut-off values for de novo OAB because post-PUFR also decreased in other groups and the current study was statistically under-powered (insufficient patient numbers in each group). Further studies with prospective designs and large cohorts are needed to confirm our findings.

## 5. Conclusions

We found that post-PUFR significantly decreased in the de novo OAB group, and it was the only significant predictor of de novo OAB after the Remeex procedure in women with SUI due to ISD. Our results show that a decrease in PUFR after the Remeex procedure is a promising metric indicating that the patient should be screened for de novo OAB. This metric can be easily and noninvasively determined.

## Figures and Tables

**Table 1:** Comparison between women according to preoperative and postoperative OAB after the Remeex procedure for stress urinary incontinence with intrinsic sphincter deficiency (data are means ± standard deviation).

**Potential predictive variable**		**Total**	**Group 1**	**Group 2**	**Group 3**	**Group 4**	***P* value** **∗**
		*n*=130	*n*=46	*n*=15	*n*=25	*n*=44	
**Clinical variables**							
Age (year)		59±11	57±10	60±15	55±11	62±9	0.036_ _^a^
BMI (kg/m^2^)		24.6±3.3	24.4±2.8	24.9±4.2	24.6±3.1	24.7±3.6	0.979_ _^a^
Diabetes	Present	18	3	2	4	9	0.286_ _^b^
	Absent	112	43	13	21	35	
Multiparity	Present	61	25	4	8	24	0.080_ _^b^
	Absent	69	21	11	17	20	
Menopause	Present	93	33	9	17	34	0.605_ _^b^
	Absent	37	13	6	8	10	
Previous hysterectomy	Present	31	9	6	7	9	0.371_ _^b^
	Absent	99	37	9	18	35	
Previous incontinence surgery	Present	32	7	3	9	13	0.196_ _^b^
	Absent	98	39	12	16	31	
Previous POP surgery	Present	10	3	1	3	3	0.847_ _^b^
	Absent	120	43	14	22	41	
Pyuria	Present	9	1	0	3	5	0.167_ _^b^
	Absent	121	45	15	22	39	

**Urodynamic variables**							
Pre-PUFR (ml/sec)		25.1±6.3	24.7±6.2	25.7±2.9	23.0±5.8	26.6±7.4	0.104_ _^a^
Pre-PVR (ml)		14±25	15±25	7±11	11±18	18±32	0.306_ _^a^
MCC (ml)		297±46	297±61	305±14	294±52	296±31	0.799_ _^a^
VLPP (cm H_2_O)		45±10	45±9	47±9	44±12	43±9	0.381_ _^a^
MUCP (cm H_2_O)		57±25	58±27	51±19	53±23	61±25	0.516_ _^a^
PdetQmax (cm H_2_O)		18±9	19±9	20±11	16±8	17±8	0.415_ _^a^
DO	Present	26	9	1	9	7	0.105_ _^b^
	Absent	104	37	14	16	37	
Post-PUFR (ml/sec)		20±8	20.7±8.1	13.2±3.8	22.3±6.6	21.7±8.3	0.001_ _^a^
Post-PVR (ml)		43±67	29±40	97±113	40±67	41±63	0.005_ _^a^

BMI = body mass index; POP = pelvic organ prolapse; pre-PUFR = preoperative peak urinary flow rate; pre-PVR = preoperative post-void residual; MCC = maximal cystometric capacity; VLPP = Valsalva leak point pressure; MUCP = maximal urethral closing pressure; PdetQmax = detrusor pressure at peak urinary flow; DO = detrusor overactivity; post-PUFR = peak urinary flow rate at one month after the Remeex procedure; post-PVR = post-void residual at one month after the Remeex procedure.

*∗p*<0.05 was considered as statistically significant.

^a^Kruskal-Wallis test.

^b^ Chi-square test.

**Table 2:** Pair wise comparisons of age, post-PUFR, and post-PVR among four groups.

**Variable**	**Group 1**	**Group 2**	**Group 3**	**Group 4**	***P* value** **∗**_ _^**a**^	***P* value** **∗**_ _^**b**^					
						**G1 vs G2**	**G1 vs G3**	**G1 vs G4**	**G2 vs G3**	**G2 vs G4**	**G3 vs G4**
Age	57±10	60±15	55±11	62±9	0.036	0.756	0.329	0.036	0.543	0.300	0.004
post-PUFR	20.7±8.1	13.2±3.8	22.3±6.6	21.7±8.3	0.001	0.001	0.402	0.654	0.001	0.000	0.689
post-PVR	29±40	97±113	40±67	41±63	0.005	0.001	0.287	0.104	0.009	0.011	0.807

Post-PUFR = peak urinary flow rate at one month after the Remeex procedure; post-PVR = post-void residual at one month after the Remeex procedure.

*∗p*<0.05 was considered as statistically significant.

^a^ Kruskal-Wallis test.

^b^ Post-hoc least significant difference test.

**Table 3:** Logistic regression analysis of predictive factors for overactive bladder at 6 months after the Remeex procedure in women with stress urinary incontinence due to intrinsic sphincter deficiency.

Predictive factor*∗*	Univariate			Multivariate		
	*P*	OR	95% CI	*P*	OR	95% CI
Age (year)	0.648	1.012	0.963-1.063	0.365	0.974	0.921-1.031
Post-PUFR (ml/sec)	0.001	0.821	0.734-0.919	0.002	0.823	0.727-0.931
Post-PVR (ml)	0.007	1.008	1.002-1.015	0.121	1.005	0.999-1.012

*∗*All parameters were analyzed as continuous variables per unit.

OR = odds ratio; CI = confidence interval; post-PUFR = peak urinary flow rate at one month after the Remeex procedure; post-PVR = post-void residual at one month after the Remeex procedure.

## Data Availability

The data used to support the findings of this study are available from the corresponding author upon request.

## Abbreviations and Acronyms

BOOI: Bladder outlet obstruction index
DO: Detrusor overactivity
ISD: Intrinsic sphincter deficiency
MCC: Maximal cystometric capacity
MUCP: Maximum urethral closure pressure
OAB: Overactive bladder
PdetQmax: Detrusor pressure at peak urinary flow
post-PUFR: PUFR one month after surgery
post-PVR: PVR one month after surgery
PUFR: Peak urinary flow rate
PVR: Postvoid residual
SUI: Stress urinary incontinence
TVT: Tension-free vaginal tape
TVT-O: Transobturator tension-free vaginal tape
UDS: Urodynamic study
VLPP: Valsalva leak-point pressure.
